# Feasibility and acceptability of a whole-school social-marketing intervention to prevent unintended teenage pregnancies and promote sexual health: evidence for progression from a pilot to a phase III randomised trial in English secondary schools

**DOI:** 10.1186/s40814-022-00971-y

**Published:** 2022-03-04

**Authors:** R. Ponsford, S. Bragg, R. Meiksin, N. Tilouche, L. Van Dyck, J. Sturgess, E. Allen, D. Elbourne, A. Hadley, M. Lohan, C. H. Mercer, G. J. Melendez Torres, S. Morris, H. Young, R. Campbell, C. Bonell

**Affiliations:** 1 Department of Public Health, Environments & Society, London School of Hygiene and Tropical Medicine, 15-17 Tavistock Place, London, WC1H 9SH UK; 2 Department of Education, Practice and Society, University College London, 20 Bedford Way, WC1H 0AL London, UK; 3 Clinical Trials Unit, London School of Hygiene and Tropical Medicine, Keppel Street, London, WC1E 7HT UK; 4 Teenage Pregnancy Knowledge Exchange, University of Bedfordshire, University Square, Luton, LU1 3JU UK; 5 School of Nursing and Midwifery, Queens University Belfast, University Road, Belfast, BT7 1NN UK; 6 University College London, Gower Street, London, WC1E 6BT UK; 7 University of Exeter, St Luke’s Campus, Heavitree Road, Exeter, EX1 2LU UK; 8 Department of Health and Primary Care, University of Exeter, St Luke’s Campus, Heavitree Road, Exeter, EX1 2LU UK; 9 School of Social Sciences, Cardiff University, 1-3 Museum Place, Cardiff, CF10 3BD UK; 10 School of Social and Community Medicine, University of Bristol, 39 Whatley Road, Bristol, BS8 2PS UK

**Keywords:** Sexual health, Teenage pregnancy, School intervention, Sex education, Adolescent health, Evaluation

## Abstract

**Background:**

Reducing unintended teenage pregnancy and promoting adolescent sexual health remains a priority in England. Both whole-school and social-marketing interventions are promising approaches to addressing these aims. However, such interventions have not been rigorously trialled in the UK and it is unclear if they are appropriate for delivery in English secondary schools. We developed and pilot trialled Positive Choices, a new whole-school social marketing intervention to address unintended teenage pregnancy and promote sexual health. Our aim was to assess the feasibility and acceptability of the intervention and trial methods in English secondary schools against pre-defined progression criteria (relating to randomisation, survey follow-up, intervention fidelity and acceptability and linkage to birth/abortion records) prior to carrying out a phase III trial of effectiveness and cost-effectiveness.

**Methods:**

Pilot RCT with integral process evaluation involving four intervention and two control schools in south-east England. The intervention comprised a student needs survey; a student/staff-led school health promotion council; a classroom curriculum for year-9 students (aged 13–14); whole-school student-led social-marketing activities; parent information; and a review of local and school-based sexual health services. Baseline surveys were conducted with year 8 (aged 12–13) in June 2018. Follow-up surveys were completed 12 months later. Process evaluation data included audio recording of staff training, surveys of trained staff, staff log books and researcher observations of intervention activities. Survey data from female students were linked to records of births and abortions to assess the feasibility of these constituting a phase III primary outcome.

**Results:**

All six schools were successfully randomised and retained in the trial. Response rates to the survey were above 80% in both arms at both baseline and follow-up. With the exception of the parent materials, the fidelity target for implementation of essential elements in three out of four schools was achieved. Student surveys indicated 80% acceptability among those who reported awareness of the programme and interviews with staff suggested strong acceptability. Linkage to birth/abortion records was feasible although none occurred among participants.

**Conclusions:**

The criteria for progression to a phase III trial were met. Our data suggest that a whole-school social-marketing approach may be appropriate for topics that are clearly prioritised by schools. A phase III trial of this intervention is now warranted to establish effectiveness and cost-effectiveness. Births and terminations are not an appropriate primary outcome measure for such a trial.

**Trial registration:**

ISRCTN65324176.

## Key messages regarding feasibility

- Whole-school and social-marketing interventions are promising approaches to addressing unplanned teenage pregnancy and promote adolescent sexual health. Such interventions have not been trialled in the UK and it is unclear if they are appropriate for delivery in English secondary schools.
- Our data demonstrate that it is possible to deliver a whole-school social-marketing intervention to address unintended teenage pregnancy and promote sexual health in English secondary schools with fidelity, and strong student and staff acceptability.
- It is feasible to evaluate such an intervention using a randomised controlled trial. Although linkage to births and abortions is feasible, these may not be an appropriate primary outcome for a phase III trial given declining prevalence and other concerns.

## Introduction

Despite significant declines over the last 20 years, the teenage birth rate in the UK remains higher than in other western European countries and reductions vary by region [1–3]. Although the negative impact of having a teenage birth can be overstated, even after controlling for prior disadvantage associations between teenage parenthood and long-term adverse outcomes for parents and children exist [4–7]. Teenagers are also the age group most likely to experience unplanned pregnancy with around half of conceptions to under 18s in England and Wales ending in abortion, this increasing to over 60% among those aged under 16 [2]. About 13% of young women aged under 20 having an abortion will have had one or more previously [8]. Sexually transmitted infections (STIs) also disproportionally affect young adults and cost large sums in healthcare [9, 10]. Reducing rates of unintended teenage pregnancy and improving adolescent sexual health therefore remain priorities in England.

There is good evidence that school-based relationships and sex education (RSE) is a key element in preventing unintended pregnancy and promoting sexual health [11–16]. However, existing systematic reviews suggest that curriculum interventions alone may be insufficient to produce consistent, sizeable and sustained changes in the behaviours underlying unintended teenage pregnancy and poor sexual health, and therefore population-level improvements in these outcomes [11–13, 15, 17]. Augmenting school-based interventions so that these also include whole-school elements alongside classroom-based RSE to make school environments more supportive of sexual health is one approach associated with more promising impacts on outcomes. Whole-school actions can include changes to school policies and practices to support promotion of sexual health; school-wide sexual health campaigns; involving students in planning and delivering such activities to increase reach and promote connection to school (a protective factor for avoiding teenage birth and sexual risk-taking [18–20]); parent engagement; and promoting student access to contraceptive and sexual health services. Recent reviews suggest that interventions addressing the wider school environment can have significant and sustained impacts on delaying sexual debut [21] and that school-based interventions involving such elements can increase contraception use and reduce pregnancy rates [22].

A related approach to prevention is the use of social marketing. This involves the use of commercial marketing methods for social ends including setting specific behaviour change goals; consumer research to identify needs and preferences; and segmentation and targeting consumers to tailor intervention approaches [23]. In a recent systematic review of social-marketing interventions to reduce teenage pregnancy, although heterogeneity precluded meta-analysis, narrative synthesis concluded this was a promising approach [24]. However, school-based interventions involving whole-school and social-marketing approaches to address unintended teenage pregnancy and promote adolescent sexual health have not been trialled in the UK.

Emergent evidence suggests that it is increasingly difficult for English schools to deliver public-health interventions when they are also under pressure to raise attainment and address a range of other social, emotional and health outcomes [25, 26]. Our recent research suggests that whole-school interventions involving multiple components requiring facilitation outside of class time by school staff may be particularly challenging to implement [27, 28]. It is also unclear whether a social-marketing approach to sexual health promotion, involving student-led decisions and campaigns, is acceptable to English schools, which are increasingly focused on top-down behaviour policies, student discipline and academic attainment [26, 29].

This paper reports on a pilot randomised controlled trial (RCT) of Positive Choices, the first UK study of a whole-school social-marketing intervention to prevent unintended teenage pregnancy and promote sexual health. With the Sex Education Forum (SEF) charity, and other youth and policy stakeholders, we developed an intervention, Positive Choices, informed by whole-school and social-marketing approaches previously found to be effective in preventing teenage pregnancy and promoting sexual health in the US and Australia [30–34].

**Fig. 1:**
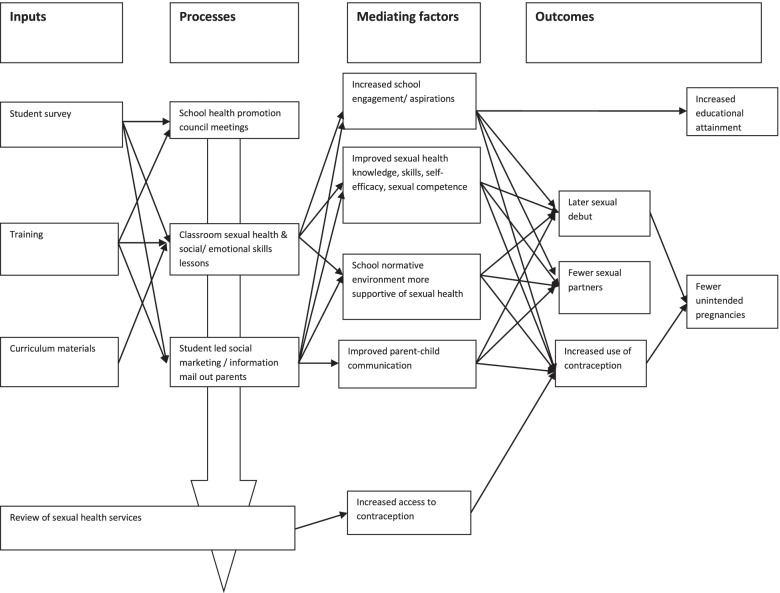
Logic model of Positive Choices

Positive Choices was designed as a manualised social-marketing intervention that aimed to reduce unintended teenage pregnancy (primary outcome) and improve sexual health. Secondary outcomes were delayed sexual debut, reduced number of partners, increased use of contraception and reduced transmission of STIs. Positive Choices was informed by social-marketing theory, addressing the ‘4Ps’ of product, place, price and promotion [35, 36]: to ‘sell’ consumers a product (education) in an accessible place (school) at low price (free to students), with promotion (campaigns, parent information) [24, 35]. Positive Choices embraces social-marketing approaches in terms of specifying prevention of teenage pregnancy as the clear goal, including consumer research (needs survey) to inform actions, and using student-led campaigns, which were to include segmenting and targeting student sub-groups [23]. Although involving whole-school elements, Positive Choices places emphasis on students in years 9 and 10 (age 13–15 years) where intervention is likely to be most relevant because proximal risk factors are manifesting [20], prevention is not too late and RSE is acceptable [33, 37, 38]. The theory of change was also informed by social influence [39] and social cognitive theory [40] in terms of addressing sexual health knowledge, self-efficacy, skills and competence, communication with parents and school-wide social norms supportive of sexual health. The intervention was further informed by the social development model [41] in terms of addressing: positive aspirations and school engagement, factors associated with teenage pregnancy and sexual risk taking [42]. The logic model is shown in Figure 1.

In line with current guidance on the development and implementation of complex interventions, prior to proceeding to a phase III trial of effectiveness, we undertook a pilot RCT of Positive Choices to assess the feasibility and acceptability of the intervention and trial methods in English schools.

This paper reports on data from the pilot study to assess whether a phase III trial of an intervention using whole-school social-marketing approaches would be feasible and promising. Criteria to progress to a phase III trial were randomisation occurred and ≥ five of six schools accepted randomisation and continued within the study; student questionnaire follow up rates were ≥ 80% in ≥ five of six schools; the intervention was implemented with fidelity in ≥ three of four intervention schools; process evaluation indicated that the intervention was acceptable to a majority of students and staff involved in implementation; and linkage of self-report and routine administrative data on pregnancies was feasible.

## Methods

### Intervention

The intervention was delivered for one academic year and comprised the following components: a student needs survey examining student knowledge, attitudes and skills related to sexual health as well as their experiences of and preferences for RSE lessons to enable each school to tailor the intervention to local priorities; a school health promotion council (SHPC) involving staff and students to review data from the needs survey to inform actions and coordinate implementation; 10 h of classroom-based RSE for year-9 students (comprising eight ‘essential’ and two ‘add-on’ lessons selected by SHPCs based on the needs data; see Table 1); student-led social-marketing campaigns around school; parent information (two homework assignments, three newsletters); and the school reviewing its own and other local sexual health services to improve provision and/or access.

**Table 1:** Positive Choices curriculum lessons

‘Essential’ lessons	‘Add on’ lessons
1. The female/male body and functions of reproductive organs	9. Pregnancy options
2. Fertility and contraception	10. Readiness for intimacy
3. Sexually transmitted infections and safer sex	11. Body image and the digital world
4. Building blocks to good relationships	12. Female Genital Mutilation
5. Consent	13. Human rights, stigma and discrimination
6. Sustaining relationships	
7. Sexual response and pleasure	
8. Pornography	

Essential modules Optional modules

School staff leading the intervention were trained by SEF in face-to-face training sessions to institute, determine the membership of and convene termly meetings of the SHPC, deliver the curriculum and facilitate student-led social marketing, supported by slides and handouts; and a manual to guide each component of the intervention. Schools also received: a report on student needs data; lesson plans and slides to guide delivery of the curriculum; a template to create three parent newsletters; two homework assignments addressing parent-child communication that were linked to lessons; and an audit tool to review and develop actions to improve provision of and access to school-based and local sexual health services. All training and materials were developed by SEF in collaboration with students and staff from one secondary school in south-east England and other youth, policy and practitioner stakeholders [43, 44].

SHPCs, comprising staff and students and facilitated by a member of school staff trained by SEF, used needs data to tailor each intervention component to local priorities and coordinate delivery. Staff chose a group of students diverse by age, gender and school engagement, guided by the manual. The needs data allowed schools to identify which of the add-on lessons would best meet their students’ needs. Teachers delivered the curriculum in various lessons and/or tutor time to students in year 9 (13–14 years). Student-led social marketing, facilitated by teachers, was led by 12–18 students per school. Campaigns used social and other media, posters and events to address healthy relationships, sexual rights, readiness for intimacy and/or access to services. Schools reviewed their own and local sexual health services to inform improvements in student access. All intervention components were delivered face-to-face on school premises.

Regarding dose, the SHPC, curriculum and student-led social-marketing training each comprised half-day sessions. SHPCs were to meet at least twice per year. Students were to receive 10 h of lessons. The intervention was funded by SEF and by schools’ contribution of staff time.

### Study design and sample size

We conducted a pilot RCT in four intervention and two control schools with integral process evaluation. The pilot focused on feasibility and no power calculation was performed.

### Participants and setting

State-funded secondary schools in south-east England were eligible to participate. We sought purposive variation in terms of high/low local deprivation and school-level educational attainment as predictors, respectively, of school need and capacity to implement.

### Recruitment, consent and surveys

Schools were invited to participate in the research via an email sent to the school’s generic email address. This method was considered appropriate as we aimed to recruit only six schools for the pilot. Response rates were recorded.

Baseline surveys were conducted before randomisation as students neared the end of year 8 (age 12/13) in June 2018. All participants deemed competent by their schools to consider consenting to participate, as well as their parents/carers, were provided with an information sheet about the study at least one week in advance of data collection. Parents/carers were asked to contact the school or research team should they wish their child not to participate. Immediately prior to any data collection, participants not already withdrawn from the research received an oral description of the study and had the chance to ask questions. All participants were asked to provide written consent. Paper questionnaires were completed confidentially in classrooms supervised by fieldworkers, with teachers remaining at the front of the class to maintain order, but unable to see student responses. Students with mild learning difficulties or limited English were supported to complete the questionnaire by fieldworkers. Absent students were surveyed by leaving questionnaires and stamped addressed envelopes with schools. Students were resurveyed 12 months later (post-intervention) in June 2019 as they neared the end of year 9 (age 13/14). Fieldworkers were blind to allocation.

### Randomisation

**Fig. 2:**
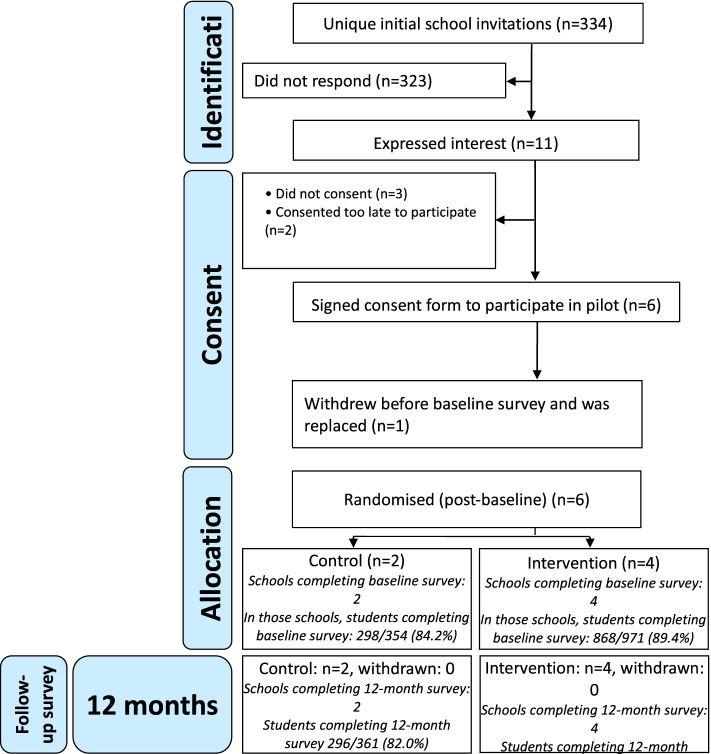
CONSORT diagram

After baseline surveys, schools were randomly allocated to intervention/control remotely by LSHTM clinical trials unit (CTU) using random number generation within two strata of school-level academic attainment. Allocation was 2:1 favouring the intervention, enabling us to pilot randomisation while maximising the number of schools in which the intervention was piloted. Control schools continued with existing sexual health-related provision. Teachers delivered RSE but the time devoted to this was much less than in Positive Choices. Neither control school had a staff/student committee that co-ordinated sexual health activities, explicitly used student-led social marketing to promote sexual health or had substantial on-site sexual health services. School randomisation and retention and student response rates and baseline and follow-up are described using a CONSORT diagram (Figure 2) [45].

### Process evaluation and measures

Process evaluation was informed by existing frameworks [46–48]. Fidelity was defined as 70%+ delivery of specified essential elements of each component. Data were collected via researcher observation and audio recording of SEF training events; surveys of trained staff; log books of school staff implementing components (checklists of the implementation of each intervention session broken down into all necessary activities); and observations of a randomly selected session per school of SHPC meetings, curriculum lessons and social-marketing meetings. Assessment of acceptability drew on student survey responses and structured elements of interviews with staff involved in implementation and focus groups with year-9 students. Quantitative data were subject to descriptive statistics indicating simple counts and percentages to describe: the fidelity with which intervention elements were delivered; student awareness and acceptability of the intervention in intervention schools; and student-reported coverage of topics in intervention and control schools. Qualitative data were subject to thematic-content analysis using techniques drawn from grounded theory such as in vivo and axial coding, and constant comparison [49].

### Data linkage

We sought to link self-report data from female students with administrative records of abortions and births up to 18 months after baseline surveys in collaboration, respectively, with the Department of Health and Social Care (DHSC) and the Office for National Statistics ONS). Linkage of such data had been previously conducted for observational studies [20] and initial discussion with ONS had established that data linkage was feasible despite the limited identifiers attached to abortion records and was consistent with DHSC and ONS guidance and data protection law.

In the case of obtaining birth data from ONS, the research team aimed to implement the following procedures to ensure the anonymity and confidentiality of data. The fieldwork team would securely transfer a password-protected dataset of female participants’ names, postcodes and dates of births (DOB), to which the CTU did not have access, linked to a unique identifier code for each participant (with the dataset not including any self-report survey data). ONS would then prepare a dataset containing unique identifier codes (but not other identifiers) linked to any births among trial participants. Having been accredited by the ONS, a member of CTU staff would then attend the ONS secure data centre to access and carry out analysis of birth data with these having been imported into the main anonymised trial dataset. A similar process was to occur to access DHSC abortion data but, in this case, using only DOB and postcode information and not participant names (because routine records of abortion data do not contain these) and would involve secure sending of each dataset rather than in-person visits.

Although ONS initially committed to providing data on births up to 18 months after baseline surveys, in the course of the project, they reported that they could only provide such data up to 11 months after baseline surveys (from July 2018 to June 2019) because of the time needed to undertake internal quality checks before releasing data.

Female survey participants were informed of this process as part of consent procedures for follow-up surveys and their specific consent for this was sought. Fieldworkers were briefed in detail about the data-linkage process and instructed to explain clearly that the researchers would not link routine data to name or share births data with others and that all data would be stored securely in line with data protection regulations. Fieldworkers were also instructed to check carefully whether consent to data linkage had been ticked on the main consent form and that, where appropriate, DOB and postcode data were complete.

### Registration

The trial was publicly registered on-line: RCTN12524938 10.1186/ISRCTN12524938 and published in an academic journal [50].

## Results

### Data collection

Of 334 schools emailed, 11 expressed interest and eight provided consent, of which six were recruited, diverse by local deprivation and school-level educational attainment. School characteristics by trial arm are presented in Table 2. Randomisation occurred and all schools were retained for the duration of the study. Response rates for student surveys were over 80% in both arms at both baseline and follow-up (Table 3).

**Table 2:** Pilot school characteristics by trial arm

School characteristics		Intervention (4 schools)	Control (2 schools)	Overall (6 schools)
School type	Academy independent of local authority	3	2	5
	Community (local authority) school	1	0	1
Academic attainment: 2017 attainment 8 score^a^ mean (SD)		47.3 (6.80)	51.1 (2.75)	48.6 (6.03)
Proportion of students entitled to Free School Meals (FSM)^a^ mean (SD)		30.25 (4.64)	23.2 (8.35)	27.9 (6.98)
School size^a^ mean (SD)		1342 (355.22)	1193 (390.50)	1292 (374.08)
Area deprivation: Income Deprivation Affecting Children Index (IDACI) score^b^ mean (SD)		0.130 (0.04)	0.183 (0.06)	0.147 (0.05)

^a^Data retrieved from www.compare-school-performance.service.gov.uk, retrieved on 6/9/19

^b^IDACI score by school post-code from imd-by-postcode.opendatacommunities.org, retrieved on 6/9/19

**Table 3:** Survey response rates

	Pilot intervention schools				Pilot control schools		Pilot total
	1	2	3	4	1	2	
Baseline student survey, *n* (%)	224 (94.1)	234 (85.4)	126 (85.1)	284 (91.3)	132 (75.9)	166 (92.2)	1166 (88.0)
Follow-up student survey, *n* (%)	214 (89.5)	220 (81.5)	133 (89.9)	296 (94.6)	142 (78.0)	154 (86.0)	1159 (87.1)
Consent to data linkage *n* (%) female students	80 (70)	83 (70)	54 (81)	127 (93)	51 (73)	58 (91)	453 (79)

All SEF training delivered at the four intervention schools was audio recorded and observed by a researcher (Table 4). The student-led social-marketing training was delivered to all schools jointly, via a conference call and slide show, and audio recorded and observed by a researcher remotely. Most staff completed satisfaction surveys following each training. In three of the intervention schools, log books for those implementing the curriculum, SHPC and student-led social marketing were completed. Researchers observed at least one curriculum lesson in each intervention school, while at least one SHPC meeting was observed in three intervention schools. Student-led social-marketing meetings were observed in all intervention schools.

**Table 4:** Completeness of other data collection

Data collection element		Pilot intervention schools				Pilot total
		1	2	3	4	
Audio recording of NCB training *n* (% of target)	SHPC	1 (100)	1 (100)	1 (100)	1 (100)	4 (100)
	Curriculum	1 (100)	1 (100)	1 (100)	1 (100)	4 (100)
	Student led social marketing	1 (100)	1 (100)	1 (100)	1 (100)	4 (100)
Trainee satisfaction surveys *n* (% of target)	SHPC	3 (75)	3 (100)	1 (100)	2 (100)	9 (89)
	Curriculum	5 (100)	0	5 (100)	4 (100)	14 (75)
	Student led social marketing	1 (100)	0	1 (100)	1 (100)	3 (75)
Log books by teaching staff *n* (% of target)	Curriculum	5 (100)	0	5 (100)	3 (100)	13 (75)
	SHPC	1 (100)	0	1 (100)	1 (100)	3 (75)
	Student led social marketing	1 (100)	0	1 (100)	1 (100)	3 (75)
Observations of one session per school *n* (% of target)	Curriculum	3 (300)	5 (500)	1 (100)	1 (100)	7 (175)
	SHPC	2 (200)	1 (100)	0	3 (300)	6 (150)
	Student led social marketing	1 (100)	0	1 (100)	2 (200)	4 (100)
Interviews with staff in intervention schools, *n* (% of target)		6 (125)	4 (100)	4 (100)	5 (125)	19 (119)

### Fidelity

#### Student needs survey

This was carried out in all four interventions schools as part of the baseline trial survey. A report summarising student needs data was provided to each intervention school at the start of the school year.

#### Training

All four intervention schools received the SEF training on running a SHPC, curriculum delivery and facilitating student-led social marketing. The SHPC training was delivered with fidelity to all four schools in the first term of the school year, with a range of 86–100% of essential items covered (Table 5). Three schools (1, 3, 4) also received the curriculum training in the first term of the school year. All three of these trainings were delivered with > 70% of essential elements covered, range 74–88%. In school 2, due to a change in head teacher and leadership of the intervention at the start of the school year, the curriculum training was delayed until the second term of the year. Time constraints imposed by the school meant that, in this training, only 50% of essential items were delivered overall. All four schools jointly received the training on social marketing in the second term of the year. According to audio recording, 94% of essential items were covered.

**Table 5:** Implementation fidelity by school and overall

Intervention component			Intervention schools				Overall/4
			1(1)	2(2)	3(3)	4(4)	
Student needs survey		Response rate (%)	94.1	85.4	85.1	91.3	4
Training	SHPC training	Attendance, *n* (sheet)	4	3	1	2	N/A
		% coverage of essential topics (observation)	91	100	100	88	4 (100)
		% coverage of essential training activities (observation)	81	100	94	86	4 (100)
		Opportunities for discussion (y/n)	Y	Y	Y	Y	4 (100)
		Overall	86	100	96	87	4 (100)
	Curriculum training	Attendance, *n* (sheet)	5	9	5	4	N/A
		% coverage of essential topics (observation)	88	66	88	76	4 (100)
		% coverage of essential training activities (observation)	80	44	88	71	4 (100)
		Opportunities for discussion (y/n)	Y	N	Y	Y	4 (100)
		Overall	83	50	88	74	3 (75)
	Student-led social marketing training	Attendance, *n* (sheet)	1	1	1	1	N/A
		% coverage of essential topics (trainer diary)	100	100	100	100	4 (100)
		% coverage of essential training activities (observation)	89	89	89	89	4 (100)
		Opportunities for discussion (y/n)	Y	Y	Y	Y	4 (100)
		Overall	94	94	94	94	4 (100)
SHPC		Number of meetings (log books)	3	2	3	3	N/A
		Topics covered (log books)	90	Missing	88	100	3 (75)
		Actions (log books)	73	Missing	81	73	3 (75)
		Opportunities for discussion (log books)	100	Missing	100	100	3 (75)
		Overall	83	Missing	81	87	3 (75)
Curriculum	Curriculum % coverage of essential topics across classes (log books)	Core lesson 1	97	Missing	100	N/A	3 (75)
		Core lesson 2	100	Missing	100	100	3 (75)
		Core lesson 3	99	Missing	100	100	3 (75)
		Core lesson 4	100	Missing	100	100	3 (75)
		Core lesson 5	100	Missing	100	100	3 (75)
		Core lesson 6	100	Missing	100	Missing	2 (75)
		Core lesson 7	100	Missing	100	100	3 (75)
		Core lesson 8	97	Missing	100	100	3 (75)
		Additional lesson 1	100	Missing	100	77	3 (75)
		Additional lesson 2	100	Missing	100	100	3 (75)
		overall % topics covered	99	Missing	100	98	3 (75)
	% coverage of essential activities (log books)	Core lesson 1	80	Missing	100	N/A	3 (75)
		Core lesson 2	97	Missing	100	100	3 (75)
		Core lesson 3	84	Missing	100	89	3 (75)
		Core lesson 4	100	Missing	100	100	3 (75)
		Core lesson 5	100	Missing	100	77	3 (75)
		Core lesson 6	100	Missing	100	Missing	2 (50)
		Core lesson 7	100	Missing	100	100	3 (75)
		Core lesson 8	96	Missing	100	100	3 (75)
		Additional lesson 1	96	Missing	100	79	3 (75)
		Additional lesson 2	97	Missing	100	100	3 (75)
		Overall % activities covered	95	Missing	100	93	3 (75)
	Opportunities for discussion (%)	Core lesson 1	100	Missing	100	N/A	3 (75)
		Core lesson 2	100	Missing	100	100	3 (75)
		Core lesson 3	100	Missing	100	100	3 (75)
		Core lesson 4	100	Missing	100	100	3 (75)
		Core lesson 5	100	Missing	100	100	3 (75)
		Core lesson 6	100	Missing	100	Missing	2 (50)
		Core lesson 7	100	Missing	100	100	3 (75)
		Core lesson 8	100	Missing	100	100	3 (75)
		Additional lesson 1	100	Missing	100	100	3 (75)
		Additional lesson 2	100	Missing	100	100	3 (75)
		Overall opportunities for discussion (%)	100	Missing	100	100	3 (75)
	Homework one set?		Y	Missing	Y	N	2 (50
	Homework two set?		Y	Missing	Y	N	2 (50)
	Overall curriculum delivery (%)		98	Missing	100	96	3 (75)
Student-led social marketing (log books)		Number of planning meetings (log books)	3	Missing	3	8	N/A
		Topics covered (log books)	100	Missing	78	67	3 (75)
		Actions (log books)	100	Missing	56	67	3 (75)
		Opportunities for discussion (log books)	100	Missing	100	100	3 (100)
		Overall	100	Missing	71	74	3 (75)
Parental information		Number of parent newsletters	2	Missing	0	1	0
Review of school sexual health services (log books)		Topics covered	83	Missing	100	83	3 (75)

#### SHPC meetings

According to staff log books, schools 1, 3 and 4 each held three SHPC meetings during the school year, with most occurring in the first term. All three achieved > 70% implementation of essential items for these meetings. No log books were received from school 2, so no fidelity assessment of this component for this school could be made. We observed two SHPC meetings at school 1, one meeting at school 2 and three meetings at school 4 to compare self-report data in log books with researcher-reported coverage of essential items. Agreement between researcher observations and staff self-reports of implementation ranged from 86 to 100%.

#### Curriculum

Fidelity of delivery of the student curriculum was high in the schools that provided log books, with all three reporting > 70% delivery of essential items across all lessons, range 96–100%. School 1 delivered the eight compulsory lessons and three add-on lessons. As agreed in advance, school 4 did not complete the first compulsory lesson as this material had already been covered in year 8 using similar SEF-produced materials. This school therefore completed seven compulsory lessons (though not providing log books for one of these) and three add-on lessons. In schools 1, 3 and 4, lessons were delivered in PSHE lessons. In school 2, the first three lessons were delivered in science classes, with (although not considered best practice for RSE) a single off-timetable day used to cover the remaining seven. Although school 2 did not return log books for the curriculum component, observation of the off-timetable day indicated 88% of essential items were covered. We observed one lesson each in schools 1, 3 and 4 and five lessons within the off-timetable day in school 2 to compare self-reported data in log books with researcher-reported coverage of essential topics. In schools 1 and 3, there was 90% agreement on essential items covered between log books and observations. Agreement could not be calculated for schools 2 and 4 as log books were not received for the lesson observed by a researcher.

#### Social marketing

According to the log books received from three schools (1, 3, 4), all implemented at least 70% of essential items in student-led social-marketing meetings and delivered a campaign. School 1 focused on accessing local services and their campaign comprised student presentations in assemblies plus quizzes and competitions for students in break-times. School 3 focused on healthy relationships and ran a poster campaign and also planned to run assemblies focused on the same topic. School 4 incorporated social marketing into year-9 performing-arts lessons and delivered a performance on healthy versus unhealthy relationships to students, staff and parents at their summer showcase. Although no log books were returned from school 2, observations indicated that several social-marketing meetings were held. A researcher observed a meeting in which a campaign was planned, focusing on social norms concerning the spreading of rumours about sexual activity among students, which was to be delivered in term 3 of the school-year.

We observed one student-led social-marketing meeting in each of the four intervention schools to compare the self-reported data in teacher log books with researcher-reported coverage of essential items. In school 1, there was 67% agreement on coverage of essential elements between log books and observations. Agreement could not be assessed for the other schools because no log books were received (school 2) or log books did not include information on the meetings observed by researchers (school 3 and 4).

#### Parent communications

Only schools 1 and 4 implemented parent newsletters. In both cases, information about Positive Choices was integrated into regular school newsletters. Information about the delivery of parent newsletters was not received from school 2 although the school lead reported that this was planned. Only intervention schools 1 and 3 set the two homework assignments. School 4 was unable to set homework for PSHE as this contravened school policy. School 2 did not report on this.

#### Sexual health services review

According to log books, schools 1, 3 and 4 reviewed sexual health services covering essential items to at least 70% fidelity. Again, no data were received from school 2.

### Acceptability

#### Student survey

Year-9 students were asked in the follow-up survey about their awareness of new RSE provision in their school (Table 6). Just under 60% reported that their school had recently been running a new RSE intervention. Of these, 80% reported that this was a good thing. Almost 90% noted that it included a new RSE curriculum and almost 60% were aware it included help for students to access sexual health services but there was lower awareness of other components. Much higher proportions of all students in intervention than control schools reported coverage of various RSE topics (Table 7), with these being a majority in intervention schools for all topics except ‘masturbation’ and ‘love’.

**Table 6:** Intervention awareness and acceptability among students in the intervention group at follow-up

Awareness measure	***N*** (%)
Report this school has recently been running a new RSE education programme	458 (59.2)
Of students reporting new programme, report that it is a good thing that the school is involved in this programme	361 (80.0)
Of students reporting new programme, report that it includes a new RSE curriculum	400 (87.3)
Of students reporting new programme, report that it includes student-led campaigns to promote sexual health	71 (15.5)
Of students reporting new programme, report that it includes information for parents	135 (29.5)
Of students reporting new programme, report that it includes help for students to access sexual health services	268 (58.5)
Of students reporting new programme, report that it includes student involvement in decisions	116 (25.3)

**Table 7:** Coverage of RSE topics by group

Coverage		Control	Intervention
		*N* (%)	*N* (%)
Sex education covers well/very well	Body	151 (56.6)	556 (69.6)
	Parts of genitalia	135 (50.6)	543 (68.0)
	Conception	154 (57.7)	638 (79.8)
	Contraception options	160 (59.9)	670 (83.9)
	STIs	99 (37.1)	586 (73.3)
	Condom use	69 (25.8)	548 (68.6)
	Safer sex	31 (11.6)	347 (43.4)
	Abusive relationships	73 (27.3)	549 (68.7)
	Sex education covers help for abuse well/very well	129 (48.3)	508 (63.6)
	Sexual consent	166 (62.2)	662 (82.9)
	Sexual pleasure	66 (24.7)	480 (60.1)
	Masturbation	40 (15.0)	379 (47.4)
	Law on pornography	78 (29.2)	543 (68.0)
	Law and naked image sharing	122 (45.7)	551 (69.0)
	Resisting pressure	143 (53.6)	600 (75.1)
	Media and body image	115 (43.1)	495 (62.0)
	FGM	21 (7.9)	435 (54.4)
	Love	66 (24.7)	395 (49.4)
	Managing conflict	67 (25.1)	431 (53.9)
	Pregnancy options	61 (22.8)	483 (60.5)
	Readiness for intimacy	55 (20.6)	412 (51.6)
	Sexual rights	69 (25.8)	431 (53.9)

#### Staff survey and interviews

Satisfaction surveys of staff undergoing training by SEF indicated that all three training were received very positively by participants, being rated as excellent or good by all. All participants reported that they felt either confident or very confident to deliver the relevant intervention component after training and that they would recommend the training to colleagues. Of staff we interviewed who had been involved in implementing Positive Choices, 18/19 indicated positive views about the acceptability of the intervention in their school. These staff indicated that Positive Choices was feasible and acceptable in their school. All four lead staff stated that they would use the Positive Choices teaching materials again. In all four schools, other aspects of the programme, such as the student-led social marketing and/or SHPC, were planned to continue the following year. Seventeen (94%) of the 18 classroom teachers interviewed stated that they enjoyed teaching most of the materials but two (11%) expressed concerns that they were personally not best placed to have done so. Four (22%) also queried the age-appropriateness of the materials.

### Data linkage

Across schools, consent to checking births and abortions records ranged from 70 to 93%. Those that did object usually cited concerns over confidentiality or the rationale for collecting such personal or what they considered useless (because they were not yet sexually active) data. A total of 453 (79%) of female students responding to the survey provided postcode and/or DOB information at follow-up, with 9% of these providing incomplete or invalid data.

In terms of births, name, postcode and DOB data were matched to ONS births registration data for the period 1 July 2018–1 June 2019. There were no births among the trial cohort based on exact matching of name, DOB and postcode. Focusing on the subset of the births data where mother’s year of birth was equivalent to that in the survey data (2004/5) produced 81 matches. Focusing separately on given/first and family/second names and running searches for matching values across both data sets resulted in 44 records that did not match either to a first name or family name, 26 that matched to a first name only and 10 that matched to a family name only and one to a family name and first name. For the one record that matched on family name and first name, neither the date of birth nor the postcode matched the participant data. It was determined, therefore, that there were no probable matches in our cohort.

In terms of abortions, postcode and DOB information were matched by the Department of Health and Social Care (DHSC) with abortions data in the Abortion Notification System (ANS) for the period 1 June 2018 to 9 October 2019. There were no abortions among the trial cohort based on exact matching of DOB and postcode. There were 166 cases in our dataset with an exact match on postcode only. Some of these matched more than once because there was more than one record with the same postcode in the ANS, giving a total of 235 individual matches on postcode only.

However, none of these matches in the ANS had a birth year of 2004 or 2005, as was the case for all females in our cohort and none of the DOBs contained in our dataset were considered close enough to those in the ANS to constitute a possible match. There were no matches on DOB alone.

A search of partial matches based on postcode sector, combing a postcode area, a postcode district and a single character indicating the location’s inward code (e.g. YO23 2), revealed 428 cases that matched at least one case in the ANS. There were a total of 9,784 individual abortion records in the ANS database that matched on postcode sector. Thirty-eight cases (or 47 matches due to the same postcode sector appearing more than once in the ANS data) had year of birth of either 2004 or 2005, for the females in our cohort. Nineteen of these 47 matched our data on year of birth, but none matched on day or month. From this, we concluded that there were also no probable partial matches in our cohort.

## Discussion

### Summary of findings

All progression criteria, relating to randomisation, survey follow-up, intervention fidelity and acceptability, and linkage to birth/abortion records, were met. In terms of randomisation, all schools recruited remained in the trial and accepted randomisation. There was a low response rate from schools but this was expected given our cheap, low-intensity approach focused on recruiting only six schools. A more intense strategy involving phone calls to schools may be required in a phase III trial involving more schools. In terms of follow-up, there were over 80% response rates at baseline and follow-up, and in each trial arm. Linkage to birth/abortion records was feasible and acceptable to just under 80% of female participants, although no births or abortions occurred among participants.

In terms of intervention fidelity, overall, the target of 70%+ implementation of essential elements in three of the four schools was met. The needs survey, SHPC and student-led social-marketing training were implemented with fidelity in all four schools. The curriculum training, SHPC meetings, curriculum lessons, student-led social-marketing meetings and sexual health services review were implemented with fidelity in three out of four schools.

In terms of acceptability, a high proportion of students reporting awareness of the intervention reported that this was acceptable. Staff reported positive views about Positive Choices. Much higher proportions of students in intervention than in control schools reported comprehensive coverage of most RSE topics.

The criterion that linkage of self-report and routine administrative data on pregnancies is feasible was also met. There were no births or abortions among the trial cohort based on exact or approximate matching.

### Limitations

Limited data received from implementation in school 2 meant that a rigorous assessment of overall fidelity for this school could not be made. There may have been some participation bias in the sample as schools were largely self-selecting. The evaluation was a pilot RCT and therefore did not aim to assess intervention effectiveness.

### Implications for research and policy

This pilot study provides the first evidence that a whole-school social-marketing intervention to promote sexual health is feasible to deliver with good fidelity, reach and acceptability in English secondary schools.

Our study also suggests that interventions specifically utilising social-marketing approaches (needs survey, student participation, student-led social marketing) to address sexual health in English schools are feasible and acceptable. Finally, in contrast to some previous studies [51], this research suggests that delivery of RSE lessons by teachers can be acceptable to students but also reaffirms the need to be attentive to selecting which staff should deliver these [52, 53]. Given that RSE is becoming compulsory in English secondary schools and current guidance advocates the use of whole-school approaches [54], our research makes a timely contribution. Following refinements based on staff and student feedback, a phase III trial of this intervention is now warranted to establish effectiveness and cost-effectiveness.

The pilot study found that trial methods were feasible but suggests several ways in which they could be refined for a phase III RCT. Routine data on births and abortions, though feasible to collect (albeit with consent to data linkage slightly below 80% among female trial participants), do not make for an appropriate primary outcome. There were no births or abortions among the trial cohort drawing on routine data. Prevalence of teenage pregnancy is now so low nationally that powering a primary analysis based on births and abortions would require a very large sample size. The under-18 conception rate in England and Wales has reduced by 62% since 1998. In 2017, the conception rate was 17.9 per thousand women aged 15 to 17 years, a 14.7% decrease from 21.0 in 2015. This fall consists of reductions in births (decreased by 15.5%) and abortions (decreased by 13%) since 2015 [55].

Focusing on births and abortions as the primary outcome is also inappropriate given the much broader aims of RSE. These include avoidance of STIs, as well as preparing young people, whatever their sexual orientation and gender identity, for healthy, communicative and non-abusive relationships and consensual, safe and pleasurable sex at a point when individuals feel ready for this. We recommend that a future phase III trial instead focuses on sexual competence at first sex as its primary outcome measure. This measure has been developed through the National Survey of Sexual Attitudes and Lifestyles (Natsal) in terms of the following at first sex: use of contraception; autonomy of decision to have sex (not due to drunkenness, external pressure etc.); partners being equally willing to have sex; and individuals judging it to have been the ‘right time (not reporting they should have waited longer etc.)’ [56, 57]. Most young people in Britain do not report competence at first sex [57]. Lack of competence at first sex is strongly associated with increased risk across adolescence and adulthood of unplanned pregnancy; sexually transmitted infection (STI) diagnosis (ever) among young women; experiencing non-volitional sex (ever); and sexual function problems (in the last year) [56]. Lack of competence at first sex is a stronger predictor of adverse sexual health outcomes than age of sexual debut alone, particularly among young women and particularly for a broader range of outcomes including non-volitional sex [56]. This measure was piloted in our study and found to have high completion (88%) and inter-item reliability (ordinal alpha = 0.74).

## Data Availability

The dataset supporting the conclusions of this article are available on request.
